# Wireless Relay Selection in Pocket Switched Networks Based on Spatial Regularity of Human Mobility †

**DOI:** 10.3390/s16010094

**Published:** 2016-01-18

**Authors:** Jianhui Huang, Xiuzhen Cheng, Jingping Bi, Biao Chen

**Affiliations:** 1 Institute of Computing Technology, Chinese Academy of Sciences, Beijing 100191, China; bjp@ict.ac.cn; 2 Department of Computer Science, The George Washington University, Washington, DC 20052, USA; cheng@gwu.edu; 3 Department of Computer Information Science, University of Macau, Macau 999078, China; bchen@umac.mo

**Keywords:** pocket switched networks, home-based relay selection, visiting frequency, human mobility

## Abstract

Pocket switched networks (PSNs) take advantage of human mobility to deliver data. Investigations on real-world trace data indicate that human mobility shows an obvious spatial regularity: a human being usually visits a few places at high frequencies. These most frequently visited places form the home of a node, which is exploited in this paper to design two HomE based Relay selectiOn (HERO) algorithms. Both algorithms input single data copy into the network at any time. In the basic HERO, only the first node encountered by the source and whose home overlaps a destination’s home is selected as a relay while the enhanced HERO keeps finding more optimal relay that visits the destination’s home with higher probability. The two proposed algorithms only require the relays to exchange the information of their home and/or the visiting frequencies to their home when two nodes meet. As a result, the information update is reduced and there is no global status information that needs to be maintained. This causes light loads on relays because of the low communication cost and storage requirements. Additionally, only simple operations are needed in the two proposed algorithms, resulting in little computation overhead at relays. At last, a theoretical analysis is performed on some key metrics and then the real-world based simulations indicate that the two HERO algorithms are efficient and effective through employing only one or a few relays.

## 1. Introduction

### 1.1. Wireless Relay Selection in PSNs

Pocket switched networks (PSNs) make use of the mobility of human to provide opportunistic communications for mobile devices. PSN communications can exploit both local and global connectivities. The former refers to the direct communications among mobile devices, and the latter needs the assistance from infrastructures such as access points (APs), base stations, and so on.

The necessities of developing PSNs come from two major drives. First, PSNs can employ unused wireless resources to realize green opportunistic communications. Currently, mobile devices are connected mainly via Internet Protocol (IP)-centric networking. Only exploiting this mode hardly utilizes fully the local wireless resources. In PSNs, mobile devices (such as laptops, personal digital assistants, and mobile phones)can carry more than one wireless interface such as Bluetooth and WiFi. Whenever two mobile devices happen to come into each other’s wireless range due to the mobility of their users, information exchanges can be realized. Second, PSNs might be the only communication vehicle in some situations. The wireless coverage of IP-centric networks are limited and such networks are fragile when natural disasters or other failures happen. In such cases, PSNs should be used to connect the islands of various IP-centric networks. Overall, PSNs can be viewed as an important means to complement traditional networks for realizing universal wireless connectivity [1,2,3,4].

Actually, PSNs is a kind of special delay/disruption tolerant networks (DTNs) [5]. They have traits of DTNs, including restricted network capacity, limited energy and storage, as well as intermittent links, which require the applications in PSNs to be delay/disruption tolerant and hence traditional IP protocols are not applicable. As a result, the communications in PSNs should adopt the store-carry-forward mode. In such networking, mobile nodes (In this paper, a node refers to a person carrying mobile device.) should act as relays to communicate opportunistically with others. As a result, relay selection is a key factor affecting the efficiency of PSNs.

Whether a relay is good or not in PSNs is decided by the reachability from the relay to the destination. However, to measure such reachability is challenging due to the time-varying traits of PSNs. Furthermore, the power of each relay in a PSN is usually limited and hence the burden of a relay increases rapidly with the growing number of mobile devices in a PSN. As a result, an effective and efficient relay selection mechanism should be light weight and scalable. To that aim, the relay selection mechanism should be designed according to the following requirements: (i) no global knowledge needed; (ii) infrequent information updating; and (iii) low burden on relays. As depicted in Section 2, the existing relay selection mechanisms cannot meet the above requirements simultaneously.

This paper proposes two HomE based Relay selectiOn (HERO) algorithms (a basic HERO and an enhanced HERO) for PSNs taking advantage of the spatial regularity of human mobility [1]. These two algorithms inject single data copy in the network at any time.

In the basic HERO, only the first node contacted by the source and its home overlaps destination’s home is selected as relay, while the enhanced HERO keeps finding the optimal relay with a hipgh visiting probability to the destination place. Both algorithms satisfy all above three design requirements. In the two algorithms, a concept of home is introduced, which indicates a set of places visited by a node frequently. Because there is high probability that a node returns home, the node can meet the destination with a high probability if its home includes the place in which the destination resides. Our HERO algorithms just select such a node as the relay for enhancing the delivery ratio.

### 1.2. Our Contributions

Our HERO algorithms are light-weighted and scalable because:

1. No global networking status information is kept on each node in HEROs, resulting in low storage requirement and communication overhead. Existing relay selection algorithms such as [6,7,8,9] require all nodes to keep and update the information of all other nodes in the network, and [10,11] require all nodes to maintain the global routing table and refresh it when needed. While in HERO, a relay is selected based on the local information, *i.e.*, home, resulting in little burden.
2. Less information updating. In HEROs, every node should maintain its home information, which is relatively stable in a time-varying PSN and no frequent information is required to update. In some DTN relay selection algorithms [10,11], each node constructs the global routing table from its local observations. Thus, the integrity, correction and consistency of the global routing table can not be guaranteed. In such cases, the relays may act according to different or even incorrect routing tables.
3. HERO is distributed in nature. Many existing PSN relay selection mechanisms [12,13,14] adopt a bridge to connect different communities, and such a bridge will be the potential communication bottleneck. While HERO requires no central node, it is distributed naturally.
4. HERO is light-weighted and effective. HERO only needs simple operations for relay selection. As a result, very low computation overhead is induced. Moreover, there is no sophisticated human mobility model is applied in HERO. For example, no strict repetitive motions of nodes is required like [15], and there is no distribution assumption of the inter-contact time between two nodes. The effectiveness and practicability is verified by our performance simulation based on the mobility trace data from Dartmouth College.

It should be noted that though the HERO algorithms are proposed for single-copy based data delivery in a PSN, the relay selection principles used by the presented algorithms can be easily ported to multi-copy based data delivery strategies. In order to eliminate the influence of redundancies introduced by multiple copy data delivery and demonstrate clearly how the home contributes to the data delivery efficiency, we elaborate on the relay selection principles for single-copy based data delivery in this paper.

### 1.3. Organization

We organize the rest of the paper as follows. Section 2 presents the related work of relay selection in PSNs. Section 3 expounds the motivation of our work and then introduces the HERO model. Section 4 describes two HERO algorithms. The theoretical analysis is provided in Section 5 and the real-world trace data-based performance validation is given in Section 6. Our conclusions and future work are presented in Section 7.

## 2. Related Work

Existing algorithms can be roughly categorized into two kinds: the social-based approaches [12,13,16,17,18,19] and the location-based approaches [20,21].

The social-based schemes utilize the sociality of human beings to design routing. For example, Wu and Wang [19] converted a routing problem in a time-varying network to a static and structured feature space because it found that people with more common social features usually have high probability to meet. Guo *et al.* [22] constructs the applied user’s potential social relationships according to their verifiable attributes in a privacy-preserving way.

The MobySpace [20] and HomingSpread [21] are location-based approaches. [20] selects the relay according to its similarity to the destination in terms of mobility pattern. The pattern of node mobility is defined by the probabilities that a node visits each place in a PSN. A multi-copy scheme was proposed in [21], which first spreads all copies of the information to each place the source frequently visits, where a static information holder is designated to keep the information and spread it to every mobile node visiting that place. Meanwhile, the information copies are equally split between the node with the information and the one without when they meet at the places that the source does not visit frequently. Finally, the destination obtains the information when it meets with one of the carriers.

The location-based approaches select relays according to the place visiting traits of a node itself while the social-based ones take advantage of the traits of the inter-individual relationships. Hence, the overhead of the location-based approaches keeps almost unchanged while that of the social-based approaches increases quickly when the size of the network increases, which makes the location-based approaches more scalable than the social-based ones.

HERO is a location-based relay selection mechanism for PSNs. Both MobySpace and HERO are single-copy schemes. However, each node should compute and carry the exact probability of visiting each place in MobySpace while each node only needs to keep frequently visited place(s) in HERO. Furthermore, MobySpace employs the similarity of nodes’ mobility patterns while HERO uses the distance to destination for relay selection. Our performance simulation based on the Dartmouth College mobility trace data indicates that both HERO algorithms are superior over MobySpace.

## 3. Motivation and the HERO Model

### 3.1. Design Motivation

In fact, PSNs not only have similar traits as DTNs such as time-varying topologies but also possess their own traits rooted from human mobility.

Through observing the real-world trace data [23,24,25], human mobility shows an important spacial characteristic, *i.e.*, a high degree of spatial regularity. This human mobility characteristic was introduced in our prior work [1], and here we briefly summarize it and give more illustrations to better justify our motivation. The explicit spatial regularity implies that nodes return to a few frequently visited places with a high probability. To explain this phenomenon, the mobility trace data from Dartmouth College’s wireless local area network (WLAN) [23] is employed, which records the mobility trajectory of each node in Dartmouth College.

The probabilities that each node visits its first, second, ..., 10th most frequently visited places (Due to the lack of geographical information for each node in this data set, we use the associated AP to represent the visited place of a node.) is calculated in the following four months: 21 September to 20 October and 21 October and 19 November in 2003, and 28 January to 26 February and 20 April to 19 May in 2004. The analysis of the trace data in [1] illustrates the average value of about 5346–6052 mobile nodes that roamed among 532–543 APs during each of the four months, which exhibits obvious spatial regularity of human mobility.

**Figure 1:**
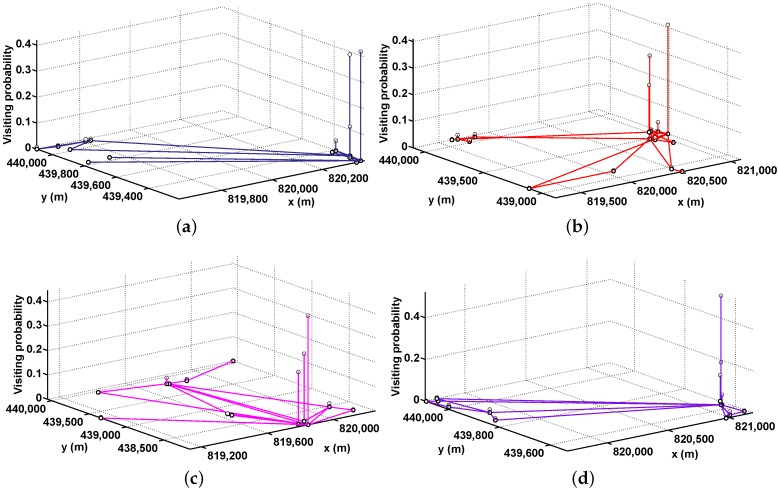
The snapshots of four mobile nodes: (**a**) from the data set 21 September 2003 to 20 October 2003; (**b**) from the data set 21 October 2003 to 19 November 2003; (**c**) from the data set 28 January 2004 to 26 February 2004; and (**d**) from the data set 20 April 2004 to 19 May 2004.

Figure 1 intuitively illustrates the characteristics of human mobility from a microcosmic perspective. It shows the snapshots of four mobile nodes selected from the four one-month data sets, where the *X* and *Y* labels are respectively the *x* and *y* coordinates of the places that the node visits, and the *Z* label is the probability that the node visits the corresponding place.

Figure 1 indicates that a human being usually visits one or some place(s) at high frequencies. Even moving over hundreds of kilometers, the probability that they come back to their most frequently visited places is high.

Based on the spatial regularity of human mobility, we propose our HERO model in the next subsection.

### 3.2. The HERO Model

**Figure 2:**
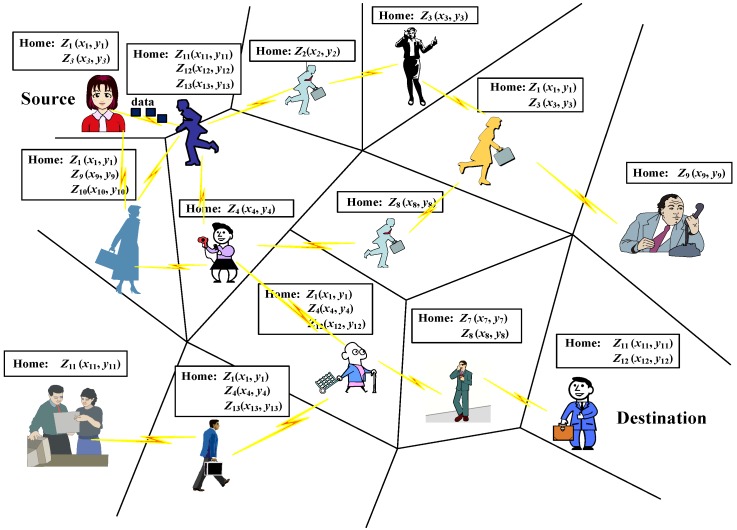
The HERO framework. In the framework, the whole network is divided into multiple non-overlapping zones which are identified by its center coordinates.

As shown in Figure 2, in HERO, the coverage area of a PSN denoted by Ω, is divided into multiple nonoverlapping zones $Z_{i}$, where $\cup Z_{i}=\Omega$ and $\cap Z_{i}=\emptyset$. Any shape is allowed for each zone $Z_{i}$, which is identified by its center coordinates $\left(x_{i},y_{i}\right)$.

As described above, human beings usually visit one or a few zones with high probabilities. These frequently visited zones are defined as the home of a node.

Home helps HERO to select relays. Thus, it is important to find the home for each node. A node can construct its home zones dynamically or statically. In this paper, two nodes within the same zone are supposed to be able to communicate with each other directly and the home information is exchanged when they contact. Relays are selected in light of the distances between their home, and the zone in which the destination resides currently. Some corresponding definitions are introduced below:

**Definition 1 (Source node).**  The source node, denoted by s, is the node that generate the data to be delivered.

**Definition 2 (Destination node).**  The destination node, denoted by d, is the node to which the data needs to be sent.

**Definition 3 (Neighbor node set).**  *The neighbor node set of node i, denoted by* $N_{i}$*, is the set of nodes that can contact node i directly.*

According to our assumption, $N_{i}$ includes all of the nodes in the same zone as node *i* and the nodes in neighboring zones that can communicate with node *i* directly.

**Definition 4 (Destination zone).**  *The destination zone, denoted by* $Z_{d}$*, is the zone in which the destination node currently resides.*

**Definition 5 (Distance between destination zone and home).**  *The distance between the destination zone* $Z_{d}$ *and the home of node i,* $H_{i}$*, is the minimum distance between any zone in* $H_{i}$ *and* $Z_{d}$*, i.e.,* $∥H_{i}-Z_{d}∥=\min\{∥Z_{i}-Z_{d}∥\;|\;Z_{i}\in H_{i}\}$.

**Definition 6 (Home node).**  *Node i is a home node of a data whose destination zone is* $Z_{d}$ *if* $∥H_{i}-Z_{d}∥=0$.

**Definition 7 (Relay node).**  A relay node can be a home node of a data if the node is selected to forward the data.

Based on the definition of the relay node, a source node is a relay node if it is a home node ofthe data.

## 4. HERO Algorithms

In this section, the basic and enhanced HERO algorithms are proposed, which store and forward a single copy of the data in PSNs all the time.

### 4.1. The Basic HERO Algorithm

In the basic HERO algorithm, if the source *s* cannot contact the destination *d* directly, a relay node will be selected from the data’s home nodes and the current relay will not forward the data to other relays until it meets *d*. The procedure is shown in Algorithm 1, where the function forward(D,i) is used to send the data *D* to relay node *i*.
**Algorithm 1** The Basic HERO Algorithm**Require:**
$N_{s}$: the neighbor node set of *s*; $Z_{d}$: the destination zone of data *D*; $H_{i}$: the home of node *i*.1:**repeat**2: Refresh $N_{s}$▹ If $d\in N_{s}$, then forward the data to *d* and exit;3: **for** each node $i\in N_{s}$
**do**4:  **if**
i=d
**then**5:   forward(D,i), **return**6:  **end**
**if**7: **end**
**for**  ▹ A home node who is the neighbor will be selected as relay; otherwise, the data will not be forwarded to other nodes;8: **if**
$∥H_{s}-Z_{d}∥>0$
**then**9:  **if**
$\exists i\in N_{s},s.t.∥H_{i}-Z_{d}∥=0$
**then**10:   forward(D,i)11:  **end**
**if**12: **end**
**if**13:**until** the data expires

### 4.2. The Enhanced HERO Algorithm

The basic HERO algorithm is simple and naive. There are many modifications that can be made to improve its data delivery performance. Here, the enhanced HERO algorithm is proposed which considers the frequencies of nodes visiting zones when selecting relay. Note that, similar to the basic HERO, only one copy of the data is maintained by the enhanced HERO algorithm in the PSN at any time. Because the visiting frequencies to a node’s home zones are different, a new definition called the intensity of visiting is introduced to depict this trait:

**Definition 8 (Intensity of Visiting).**  *The intensity of visiting of zone* $Z_{j}$ *by node i denoted by* $V_{ij}$ *indicates the times of node i visiting zone* $Z_{j}$ *in unit time.*

The single-copy and multi-relay mechanisms are adopted by the enhanced HERO algorithm based on the concept of home. In this algorithm, when relay node contacts other nodes, it will collect the information of home and the intensity of visiting from them. The core idea of the enhanced HERO is to keep looking for the optimal relay whose frequency of visiting the destination zone is higher than that of the current relay. When the source node cannot contact the destination node directly, it selects home node as relay from its neighborhood with the highest visiting intensity and forwards the data to it. If the relay node encounters another home node which has optimal visiting intensity than itself and other neighbor nodes, the data is forwarded to this home node and this home node becomes the new relay node. The procedure is shown in Algorithm 2.

Note that both of the two HERO algorithms assume potentially that the destination node resides in $Z_{d}$ during data delivery. If this assumption is invalid, *i.e.*, the destination does not stay in $Z_{d}$ when the data arriving at $Z_{d}$, our algorithms can still be available through a minor change in the data delivery strategy. In the modified version of the enhanced HERO, the data can be delivered to all the home zones of the destination according to the relay selection policy of our algorithms. In the worst case scenario that the destination does not reside in any home zone, the data can be forwarded to a static node or the infrastructure residing in the home zones of the destination, from which the destination can retrieve the data once it returns home.

It should be noted that in a large-scale sparse network, it is hard for the source to find a proper relay meeting the criterion of relay selection during a short time. In this case, the proposed algorithms will have the problem of the slow-start phase, which seriously reduces the speed of data propagation in the network, and then results in inefficient data forwarding. Hence, a simple variant of our algorithms can work like this: if the source can not encounter a suitable node meeting the given relay criterion within a given period of time, it will select the node having minimum distance between home and destination as the relay from its neighbor node set. There are many other variations that can be explored to further improve the efficiency of the HERO algorithms, which will be investigated in our future research.
**Algorithm 2** The Enhanced HERO Algorithm**Require:**
$N_{i}$: the neighbor node set of *i*; $Z_{d}$: the destination zone of data *D*; $S_{Hi}$: the home node set contacted by *i*; $V_{kj}$: the intensity of visiting of *k* to zone $Z_{j}$.1:**repeat**2: Refresh $N_{i}$    ▹ Node *i* can be the source node *s* or a relay node;3: **for** each node $k\in N_{i}$
**do**4:  **if**
k=d
**then**5:   forward(D,k), **return**6:  **end**
**if**7:  Refresh $N_{k}$8: **end**
**for**9: $S_{Hi}\leftarrow \{k\in N_{i}\;|\;∥H_{k}-Z_{d}∥=0\}$10: **if**
$S_{Hi}\neq \emptyset$
**then**    ▹ Select the neighbor node *j* who has the highest intensity of visiting to $Z_{d}$ as new relay;11:  **if**
$\exists j\in S_{Hi}\wedge \left(V_{jZ_{d}}>V_{iZ_{d}}\right)\wedge (V_{jZ_{d}}\geq V_{hZ_{d}}$ for $\forall h\in S_{Hi}\setminus \left\{j\right\})$
**then**12:   forward(D,j), **return**13:  **end**
**if**14: **end**
**if**15:**until** the time to live of data arrives

## 5. Performance Analysis and Validation

In this section, the theoretical analysis of the HERO algorithms and the corresponding numerical results for validation purpose are provided.

### 5.1. Theoretical Analysis

As introduced above, through adjusting the strategies of applying HERO algorithms, the data can be delivered to a highly mobile nodes. As a result, in the following analysis, we also assume that the destination is static during the process of data delivery. However, we would like to argue that our analysis can be easily generalized to the case when the destination is mobile.

Based on our prior assumption, two nodes within the same zone at the same time can communicate with each other directly. Thus, no relay is needed if the source and the destination reside at the same zone when the data is generated, yielding a perfect delivery ratio and a negligible delivery latency (In such a case, the delivery latency is the wireless transmission time.). Therefore our analysis focuses on the scenario where the source and the destination do not reside at the same zone when the data is generated. In such a case, the end-to-end delay, also known as data delivery latency, is the sum of the data holding time at the source node and the delivery latency by the relay(s).

**Definition 9 (Data holding time).**  The data holding time of HERO is the duration from the time when the data is generated at the source to the time when the source sends it to the first relay or the source drops it due to data expiration.

Note that if the source is a relay, the data holding time is zero.

**Definition 10 (Relay latency).**  The relay latency of data in HERO is the duration from the time when the source sends the data to the first relay to the time when the data is delivered to the destination zone.

**Figure 3:**
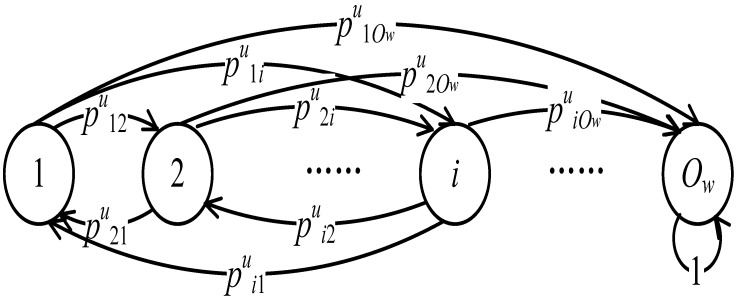
The Markov model of node mobility. Each state in the model represents the zone at which a node resides and the transition probability indicates the probability that node transfers among zones.

We assume there are *N* zones in the PSN, and the rate that nodes arrive at zone *i* follows a Poisson distribution with a parameter $\lambda_{i}$. A Markov Chain model [26], which is illustrated in Figure 3, is adopted to analyze the mobility of a relay. We call a node that moves from one zone to another in the Markov chain a transfer. A state represents the zone at which a node resides and the transition probability $p_{ji}^{u}$ is the probability that node *u* transfers from zone $Z_{j}$ to zone $Z_{i}$. Once a relay arrives at the destination zone, the data can be delivered to the destination, indicating that the data delivery is successful. Therefore, we use an absorbing state to represent the state at which the node arrives in the destination zone. Once entering the absorbing state, the node cannot leave.

Let $O_{w}$ be the absorbing state when the destination zone is $Z_{w}$ and $\Lambda_{w}$ be the set of non-absorbing states (We should not add the zones where node *u* never visits to $\Lambda_{w}$). Let $\Psi_{w}^{u}$ be the matrix of probabilities that node *u* transfers among the non-absorbing states and $\Phi_{w}^{u}$ be the vector of probabilities that node *u* transfers from the non-absorbing states to the absorbing state. Denote by $P_{w}^{nu}$ the vector of data delivery ratios after *n* transfers for node *u* when it starts from different zones. According to [26], $P_{w}^{nu}={\left(I-\Psi_{w}^{u}\right)}^{-1}\left(I-{\left(\Psi_{w}^{u}\right)}^{n}\right)\Phi_{w}^{u}$.

**Lemma 1.**  For both HERO algorithms, data carried by a relay is delivered to its destination zone with a probability of 1 if the data never expires.

**Proof.**  For both the basic and the enhanced HERO algorithms, a selected relay is always a home node of the data. Since the probability that a relay arrives at the destination zone is non-zero, all non-absorbing states sooner or later will transfer to the unique absorbing state as the data never expires. Moreover, once a node enters its absorbing state, it never leaves. Therefore, the probability that data carried by a relay is delivered to its destination is 1 if it never expires. ☐

**Lemma 2.**  *If data never expires,* ${\left(I-\Psi_{w}^{u}\right)}^{-1}≃I+\Psi_{w}^{u}+{\left(\Psi_{w}^{u}\right)}^{2}+...+{\left(\Psi_{w}^{u}\right)}^{K-1}$*, where K is an integer that makes* ${\left(\Psi_{w}^{u}\right)}^{K}≃0$*, with* 0 *being a matrix with elements of all-zero.*

**Proof.**  If data never expires, sooner or later, the absorbing state will be encountered. Thus, we have $\lim_{n\to \infty }{\left(\Psi_{w}^{u}\right)}^{n}=0$, where *n* is the number of transfers to reach the absorbing state. Let *K* be an integer that is large enough to make ${\left(\Psi_{w}^{u}\right)}^{K}≃0$. We obtain

$$\begin{array}{ccc} & & \left(I-\Psi_{w}^{u}\right)\left(I+\Psi_{w}^{u}+{\left(\Psi_{w}^{u}\right)}^{2}+...+{\left(\Psi_{w}^{u}\right)}^{K-1}\right)\\ & = & I+\Psi_{w}^{u}+...+{\left(\Psi_{w}^{u}\right)}^{K-1}-\left(\Psi_{w}^{u}+...+{\left(\Psi_{w}^{u}\right)}^{K}\right)\\ & = & I-{\left(\Psi_{w}^{u}\right)}^{K}\\ & ≃ & I \end{array}$$

Hence, ${\left(I-\Psi_{w}^{u}\right)}^{-1}≃I+\Psi_{w}^{u}+{\left(\Psi_{w}^{u}\right)}^{2}+...+{\left(\Psi_{w}^{u}\right)}^{K-1}$. ☐

**Lemma 3.**  *If data never expires,* ${\left(I-\Psi_{w}^{u}\right)}^{-1}\Phi_{w}^{u}=e$*, where* e *is a column vector with elements of all-one.*

**Proof.**  According to the proof of Lemma 2, $\lim_{n\to \infty }{\left(\Psi_{w}^{u}\right)}^{n}=0$. Thus $P_{w}^{nu}={\left(I-\Psi_{w}^{u}\right)}^{-1}\Phi_{w}^{u}$ when the number of transfers goes to infinity, which equals e according to Lemma 1. ☐

**Lemma 4.**  Assume that data never expires. Given an observation period δ, a home node with a higher visiting intensity to the destination has a higher delivery ratio than the one with a lower visiting intensity.

**Proof.**  We assume that the system undergoes *n* transfers during the observation period *δ*. In light of Lemmas 2 and 3, $P_{w}^{nu}$ can be transformed as:

$$\begin{array}{ccc} P_{w}^{nu} & = & {\left(I-\Psi_{w}^{u}\right)}^{-1}\left(I-{\left(\Psi_{w}^{u}\right)}^{n}\right)\Phi_{w}^{u}\\ & = & \left(I+\Psi_{w}^{u}+...+{\left(\Psi_{w}^{u}\right)}^{K-1}\right)\left(I-{\left(\Psi_{w}^{u}\right)}^{n}\right)\Phi_{w}^{u}\\ & = & \left(I-{\left(\Psi_{w}^{u}\right)}^{n}+...+{\left(\Psi_{w}^{u}\right)}^{K-1}-{\left(\Psi_{w}^{u}\right)}^{n+K-1}\right)\Phi_{w}^{u}\\ & = & \left(I-{\left(\Psi_{w}^{u}\right)}^{n}\right)\left(I+\Psi_{w}^{u}+...+{\left(\Psi_{w}^{u}\right)}^{K-1}\right)\Phi_{w}^{u}\\ & = & \left(I-{\left(\Psi_{w}^{u}\right)}^{n}\right){\left(I-\Psi_{w}^{u}\right)}^{-1}\Phi_{w}^{u}\\ & = & (I-{\left(\Psi_{w}^{u}\right)}^{n})e \end{array}$$

Let $a_{i}^{u}$ be the $i^{th}$ element of $\Phi_{w}^{u}$, which is the probability that node *u* visits the destination zone $Z_{w}$ from zone $Z_{i}$. Because the sum of probabilities that the node *u* visits all zones is 1 when it moves from zone $Z_{i}$, $a_{i}^{u}=1-\sum b_{ij}^{u}$, where $b_{ij}^{u}$ is the element of $\Psi_{w}^{u}$. Let $\bar{a^{u}}=\sum_{i}\frac{a_{i}^{u}}{N-1}$, which is the average probability that node *u* visits $Z_{w}$ from an arbitrary zone except $Z_{w}$.

We prove this lemma from a statistical point of view. In other words, we do not care where the node starts from. The node can start from any arbitrary zone except $Z_{w}$. Hence we replace $a_{i}^{u}$ by $\bar{a^{u}}$. Because $a_{i}^{u}=1-\sum b_{ij}^{u}≃\bar{a^{u}}$, each element of $(I-{\left(\Psi_{w}^{u}\right)}^{n})e$ is $1-{\left(1-\bar{a^{u}}\right)}^{n}$, which can be viewed as the average value of node *u*’s delivery ratio when it starts from an arbitrary zone.

According to Definition 8, if there exists another home node *v* with $V_{vw}>V_{uw}$, $\bar{a^{v}}>\bar{a^{u}}$, which makes $1-{\left(1-\bar{a^{v}}\right)}^{n}>1-{\left(1-\bar{a^{u}}\right)}^{n}$. That is, node *v* with a higher $V_{vw}$ has a higher average delivery ratio than node *u*. ☐

**Theorem 1.**  *Assume that the data never expires. Given the source zone* $Z_{i}$ *and the destination zone* $Z_{w}$*, the average delivery ratio in the enhanced HERO is higher than that in the basic HERO for a finite interval starting from the time when the data is generated.*

**Proof.**  The proof follows Lemma 4 directly. ☐

The following three theorems investigate the data holding time and relay latency of both HERO algorithms.

**Theorem 2.**  *Given the source zone* $Z_{i}$ *and the destination zone* $Z_{w}$*, the expected data holding time is* $\frac{1-e^{-\lambda_{i}\pi_{w}T}\left(1+\lambda_{i}\pi_{w}T\right)}{\lambda_{i}\pi_{w}}+e^{-\lambda_{i}\pi_{w}T}T$ *for both HERO algorithms, where T is the data lifetime and* $\pi_{w}$ *is the probability that the zone* $Z_{w}$ *belongs to the home of any node.*

**Proof.**  Because the rate at which nodes arrive at zone $Z_{i}$ follows a Poisson distribution with a parameter $\lambda_{i}$ and the probability that the zone $Z_{w}$ is a zone of any node’s home is $\pi_{w}$, the time interval that a home node arrives at zone $Z_{i}$ follows a negative exponential distribution with a probability density function $f_{i}\left(t\right)=\lambda_{i}\pi_{w}e^{-\lambda_{i}\pi_{w}t}$. Therefore the expected data holding time becomes $\int_{0}^{T}tf_{i}\left(t\right)dt+\int_{T}^{+\infty }Tf_{i}\left(t\right)dt=\int_{0}^{T}tf_{i}\left(t\right)dt+T\left(1-\int_{0}^{T}f_{i}\left(t\right)dt\right)=\frac{1-e^{-\lambda_{i}\pi_{w}T}\left(1+\lambda_{i}\pi_{w}T\right)}{\lambda_{i}\pi_{w}}+e^{-\lambda_{i}\pi_{w}T}T$ for a data that expires after *T*. ☐

Note that when *T* approaches to infinity, which corresponds to the case that the data never expires, the average data holding time becomes $\frac{1}{\lambda_{i}\pi_{w}}$.

**Theorem 3.**  *Given the source zone* $Z_{i}$ *and the destination zone* $Z_{w}$ *in the basic HERO algorithm, if node u is selected as the relay, the average relay latency is* $\sum_{j\in \Lambda_{w}}c_{ij}^{u}T_{j}^{u}$*, with* $c_{ij}^{u}$ *being the element of* $C_{w}^{u}={\left(I-\Psi_{w}^{u}\right)}^{-1}$*, and* $T_{j}^{u}$ *being the average time that the relay u resides at zone* $Z_{j}$.

**Proof.**  Let $C_{w}^{u}={\left(I-\Psi_{w}^{u}\right)}^{-1}$. According to [26], the element $c_{ij}^{u}$ of $C_{w}^{u}$ is the number of times relay *u* visits state $j\in \Lambda_{w}$ before it arrives at $Z_{w}$ when it starts from state *i*. Because the number of transfers that a relay needs to go to the destination zone is the sum of the times that the relay visits all inner states starting from the source zone, the total number of transfers of relay *u* before it arrives at the destination zone $Z_{w}$ is $\sum_{j\in \Lambda_{w}}c_{ij}^{u}$ when the source zone is $Z_{i}$. Therefore the average delay is $\sum_{j\in \Lambda_{w}}c_{ij}^{u}T_{j}^{u}$, with $T_{j}^{u}$ being the average amount of time that relay *u* resides at zone $Z_{j}$. ☐

**Theorem 4.**  The average relay latency of the enhanced HERO is not longer than that of the basic HERO.

**Proof.**  According to Definition 8, the visiting intensity of a node to a zone is its frequency of visiting that zone within a unit time. Thus, the average amount of time taken by a node with a high visiting intensity to the destination zone is less than that taken by a node with a lower visiting intensity. Because the visiting intensity of a relay to the destination zone in the enhanced HERO is not lower than that of the one in the basic HERO, the average relay latency in the enhanced HERO is not larger than that in the basic HERO. ☐

### 5.2. Numerical Results

This subsection provides numerical results to validate Theorems 2 and 3, which report the expected data holding time of both HERO algorithms and the relay latency of the basic HERO, respectively. The validation of Theorems 1 and 4 are performed via the Dartmouth College trace data in Section 6 for the following reason: the results of these two theorems, which jointly claim that the enhanced HERO outperforms the basic one in terms of relay latency and data delivery ratio, rely on only the visiting intensity of a node to a zone, not the assumption that the node arrival rate within a zone follows a Poisson distribution; therefore, they are more general and should be validated via real-world trace data.

On the other hand, Theorems 2 and 3 cannot be validated via the Dartmouth College trace data for the following reasons. First, Theorem 2 assumes that the node arrival rate at a zone follows a Poisson distribution. Though this assumption is common in communication theory, the node arrival rate in the Dartmouth College trace data does not accord with any Poisson distribution based on our observation. Second, in Theorem 3, we employ the probabilities that a node transfers among different zones to analyze the relay latency of the basic HERO. The transfer probabilities have statistical significance. In other words, through counting the average value from many specimens (each specimen is a transfer route), each transfer probability can be calculated. However, for the Dartmouth College trace data, even though we can test the relay latency, we cannot compare it with the theoretical value because a transfer probability matrix of a node can not be obtained from only one specimen (because one node only has one route).

The numerical results for Theorems 2 and 3 are obtained from the following simulation settings. There are 25 zones in the network. The nodes arrive at a zone following a Poisson distribution. Without loss of generality, we set the probability that each zone belongs to any node’s home to 1/25. We randomly allocate a transfer probability matrix (TPM) for each node and let each node move according to its TPM. Through calculating the duration from when the relay receives the data to when it arrives at the destination zone, the relay latency is obtained. This test is repeated 20,000 times with different random seeds for statistical confidence.

**Figure 4:**
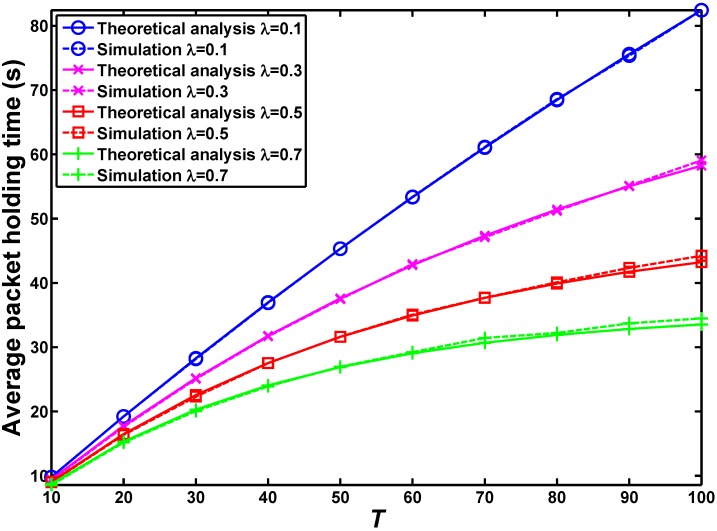
Average data holding time, which is the duration from the time when the data is generated at the source to the time when the source sends it to the first relay or the source drops it due to data expiration.

**Figure 5:**
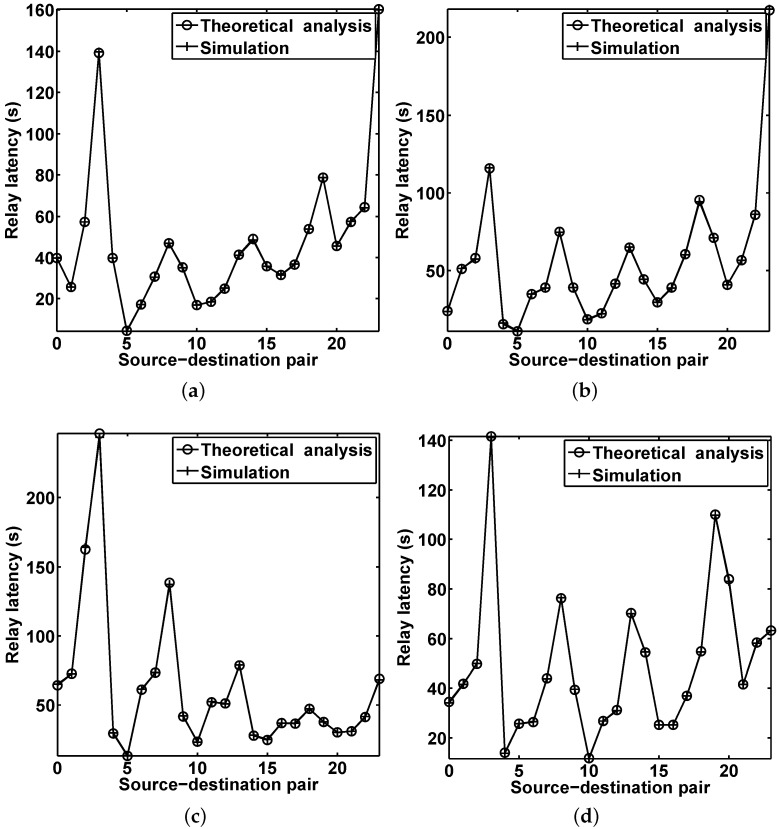
Average relay latency of the basic HERO algorithm, which is the duration from the time when the source sends the data to the first relay to the time when the data is delivered to the destination zone. (**a**) Relay 1; (**b**) Relay 2; (**c**) Relay 3; (**d**) Relay 4.

Figure 4 illustrates how the average data holding time changes with the given expiration duration *T* under different arrival rates *λ*, which is the expected number of nodes arriving at the source zone. Figure 5 reports the numerical results of the average relay latencies for four relays with different TPMs for the basic HERO algorithm. Both Figure 4 and Figure 5 indicate that our theoretical results are very close to those obtained from simulated data.

## 6. Performance Evaluation

In this section, the performance of HERO is validated based on the mobility trace data of Dartmouth College [23] from 21 September 2003 to 20 October 2003. Because the running time increases quickly as the network’s size increases, the network for performance evaluation are limited to an appropriate size. This is a common method taken by [8,10,20], in which the trace data from Dartmouth College is employed too.

In our evaluation, we do not deliberately select so called “good neighbors” [10] or “active nodes” [20] to construct the network topology. Instead, mobile nodes are selected completely randomly from the raw data. This selection criterion makes the performance of HERO and the compared schemes appear worse in our simulation than those reported in [8,10,20]. However, this does not hamper our performance validation because with limited data we care about the relative performance, not the absolute performance.

In this simulation, a zone is represented by an AP. Following literature [20], we assume that two nodes can communicate directly if they are connected to the same AP when they talk. As claimed in [20], this assumption is somewhat artificial as nodes that are attached to two different APs might be able to communicate directly if they are close enough. However, this is the best approximation we can make with the available trace data.

One-hundred mobile nodes are randomly selected as the sources and an AP is randomly assigned for each source to act as its destination. In addition, to construct the simulation scenarios of dynamic nodes, 200 mobile nodes are randomly selected, and 100 sources are added which have been already selected above to construct a 300-node scenario. After repeating the process ten times, we can obtain ten 300-node scenarios. For each 300-node scenario, 100 newly mobile nodes are added to form a 400-node scenario. Thus, we can obtain ten 400-node scenarios. Using the same method, we can construct ten 500-node and ten 600-node scenarios. For simplicity, U=x is used to denote the *x*-node scenario , where *U* is the size of network.

In this simulation, the impact of home on the HERO is firstly evaluated. Next, a performance comparison among HERO, MobySpace [20] and Epidemic [27] is given.

### 6.1. Impact of Home Size on HERO

As described above, home is the base of HERO. A node’s home are the zones where the node frequently visits. In this section, we evaluate the impact of zone number in a home, *i.e.*, the the size of a home, on HERO.

In this evaluation, the maximum number of zones belonging to a node’s home is limited. This assumption can be verified by the trace data from Dartmouth College for the period of 21 September 2003 to 20 October 2003, which shows that in the one-month period, most mobile nodes visited less than 10 zones and among the total 5543 mobile nodes, 126 of them visited more than 60 zones in the month. Table 1 shows the average home size when the maximum home size varies.

**Table 1:** The average home size *vs.* the maximum home size.

**Max.**	10	20	30	40	50	60	70	80
**Avg.**	6.46	9.33	10.68	11.36	11.79	12.06	12.24	12.36

**Figure 6:**
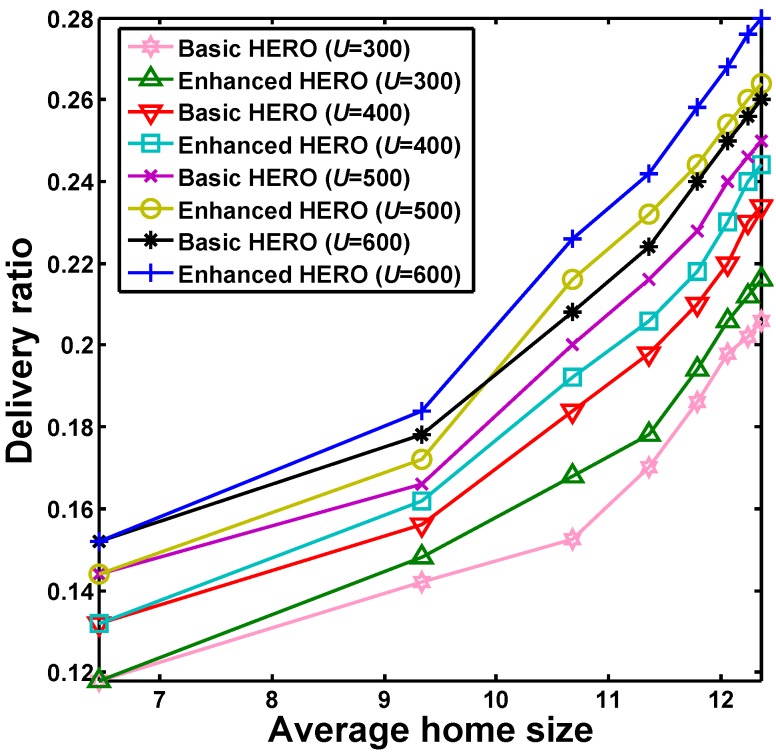
The impact of home size on the delivery ratio of HERO, which shows that delivery ratios are improved with the increase of average home size in all scenarios.

Figure 6 shows how the average delivery ratio of HERO changes with the average home size under different network size *U*. The results indicate that when the average home size increases, the average delivery ratio increases. This is because more mobile nodes may have the chance to be selected as relays when more zones are included in the homes of the nodes. Similarly, the delivery ratio increases with the network size, which results from the higher chance of successfully finding a relay as there are a higher number of candidate relays in a larger network. In addition, notice that within the observation duration of 30 days, the enhanced HERO is better than the basic HERO in term of delivery ratio, which accords with the conclusion of Theorem 1.

In the following, the impact of home size on the average relay latency and the average total data delivery latency is given. The latter is the sum of the average relay latency and the average data holding time waiting for an effective relay. From the end-to-end point of view, the average total latency makes more sense because it is the end-to-end communication delay. However, the average relay latency reflects the duration in which the network carries the data for the destination. The longer the relay latency, the higher the cost the network pays. Therefore, both the average relay latency and the average total delivery latency are effective metrics.

**Figure 7:**
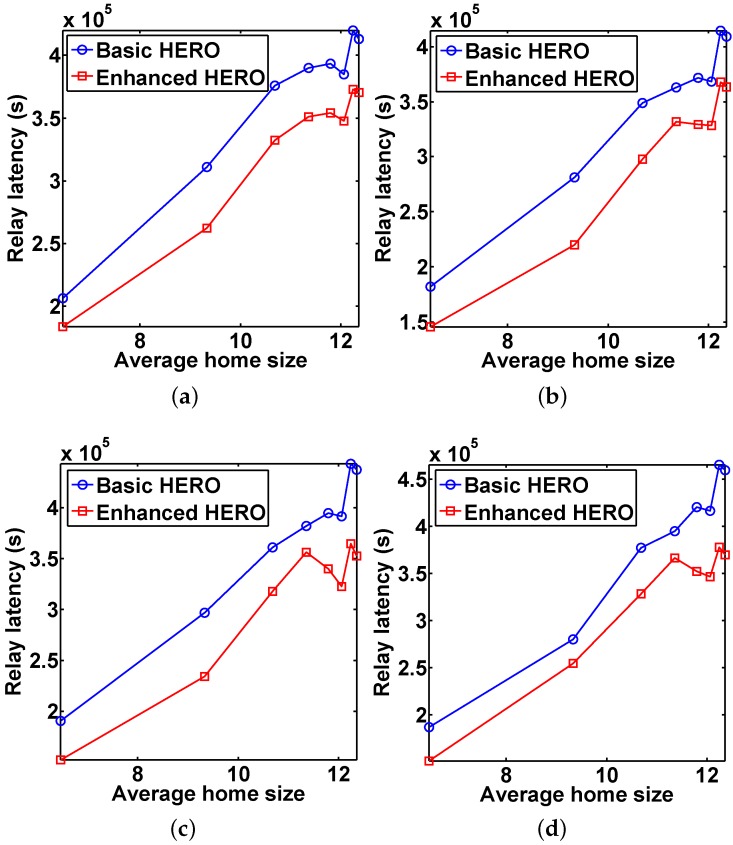
The impact of home size on the average relay latency, which shows that the relay latencies of enhanced HERO are better than those of basic HERO in all scenarios. (**a**) U=300; (**b**) U=400; (**c**) U=500; (**d**) U=600.

Figure 7 reports the average relay latency *vs.* the average home size for different *U*. The results indicate that the average relay latency of the basic HERO is higher than that of the enhanced HERO, which accords with the conclusion of Theorem 4. In addition, with the increase of the home size, the average relay latency increases. However, when the average home size equals 12.06, the average relay latency is lower than those obtained when the average home size equals 11.36, 11.79, 12.24 and 12.36.

After a further in-depth investigation, we found the reason of the above phenomena: some communication pairs that fail when the average home size is 11.36 or 11.79 succeed when the average home size is 12.06. Moreover, some of these successful communications have low relay latencies, resulting in the decreased average relay latency. It is worthy to note that the newly added successful communication pairs may increase or decrease the average relay latency.

There are two reasons helping to explain the trend of the increased average relay latencies when the average home size increases: first, the relay latencies of some of newly added successful communication pairs increase the average relay latency; second, with the increase of the average home size, the chance to select the nodes that visit the destination zone less frequently is enlarged, which prolongs the delivery delay.

**Figure 8:**
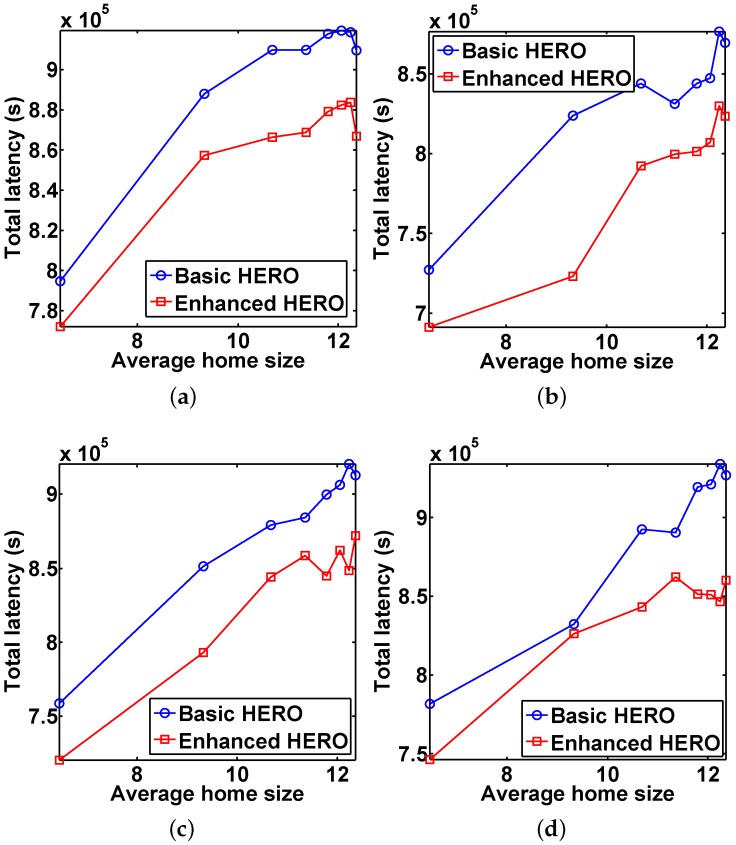
The impact of home size on the average total latency, which shows that the total latencies of enhanced HERO are better than those of basic HERO in all scenarios. (**a**) U=300; (**b**) U=400; (**c**) U=500; (**d**) U=600.

Figure 8 reports the average total delivery latency *vs.* the average home size when *U* varies. The results indicate that the average total latency of the basic HERO is higher than that of the enhanced HERO.

**Figure 9:**
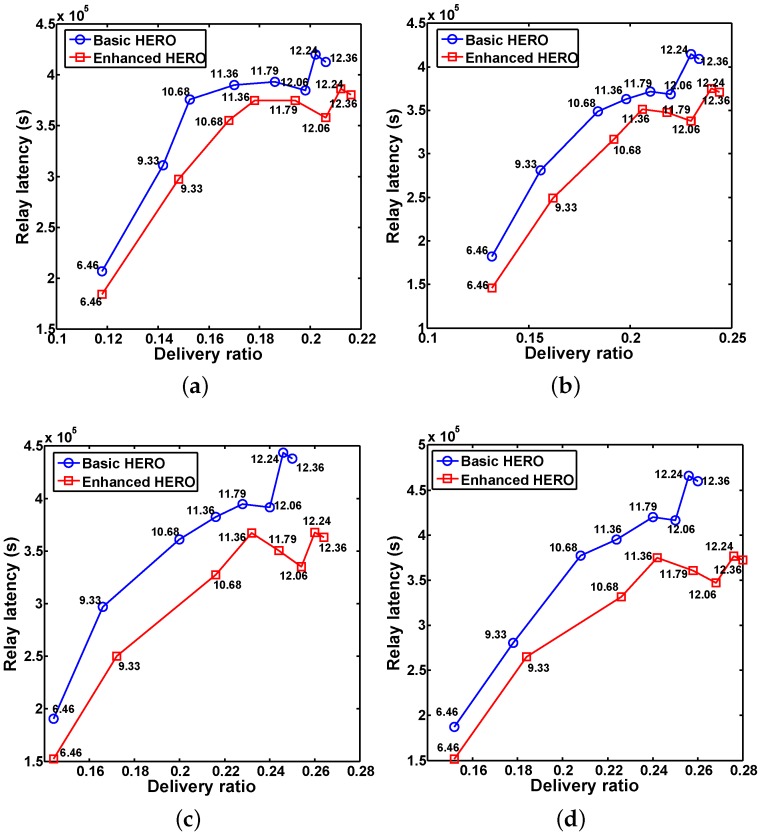
The delivery ratio and the relay latency under different average home size. The figure shows when the average home size equals 12.06, the best performance of both HERO algorithms can be obtained. (**a**) U=300; (**b**) U=400; (**c**) U=500; (**d**) U=600.

Figure 9 reports the delivery ratio *vs.* the relay latency for different home size, where the number attached to each marker is the average home size. From Figure 9, we observe that when the average home size equals 12.06, the performance of both HERO algorithms are the best. Hence in the following comparison-based study, we fix the average home size to be 12.06 for both HERO algorithms.

### 6.2. Performance Comparison

In this subsection, we compare both HERO algorithms with MobySpace [20] and Epidemic [27].

MobySpace [20] employs location information and injects single data copy to the network. MobySpace forwards the data to a node with a mobility pattern that is more similar to that of the destination. The similarity of two nodes’ mobility patterns is defined by the Euclidean distance of their MobyPoints. In Epidemic [27], a relay gives data to every newly encountered node.

**Figure 10:**
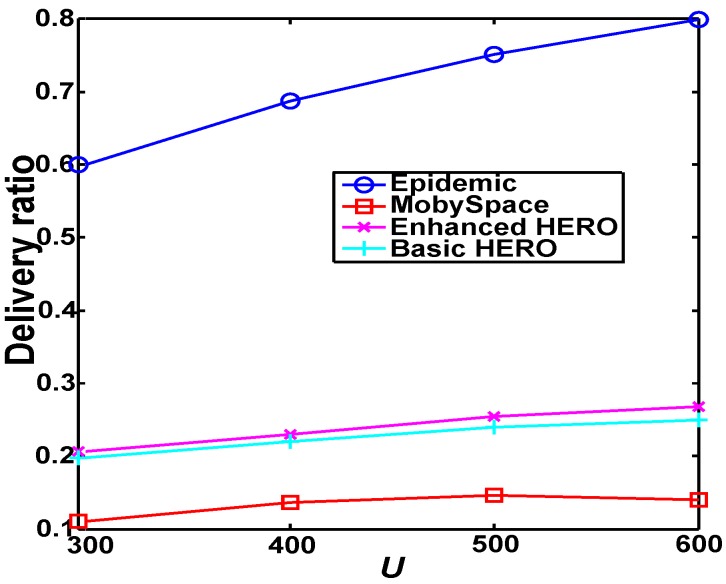
Comparison of delivery ratio. With the help of home, the delivery ratios of two HEROs are much better than that of the MobySpace.

Figure 10 shows that Epidemic is the best and MobySpace is worst in terms of the data delivery ratio. Additionally, the data delivery ratios of all algorithms except Epidemic slightly increase with the increase of network size. This is because Epidemic is actually a flooding policy, the network connectivity is increased largely with the increase of *U*. However, both HERO and Mobyspace are single-copy mechanisms, the increase of the network connectivity impacts HERO and Mobyspace slightly even though the probability of finding a good relay increases when the increase of *U*. This is why the the delivery ratio of Epidemic is improved faster than those of Mobyspace and HERO.

**Figure 11:**
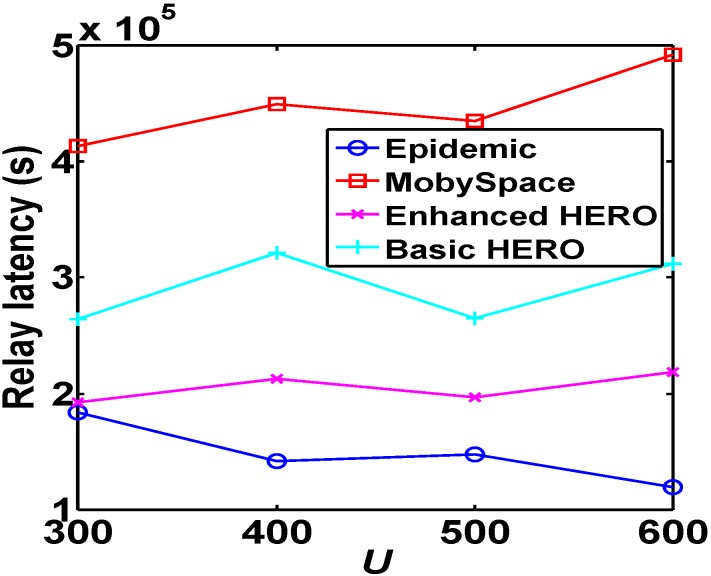
Comparison of relay latency. With the help of home zones, the relay latencies of two HERO algorithms are superior to that of the MobySpace algorithm.

**Figure 12:**
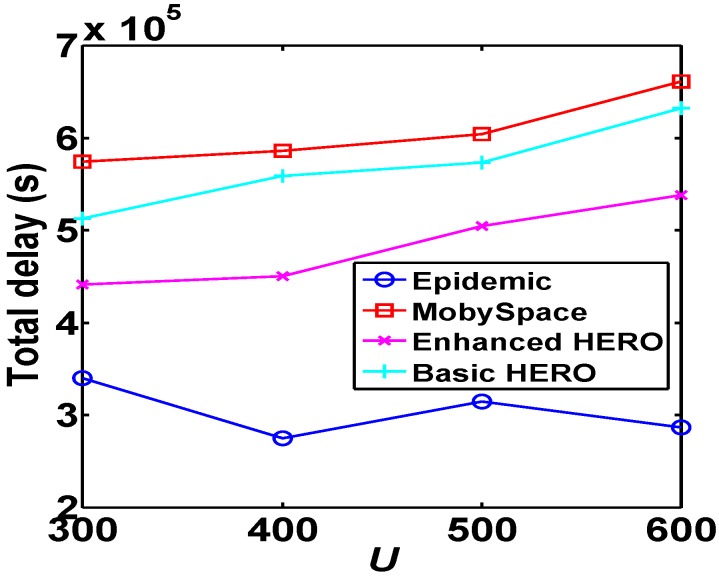
Comparison of total latency, which shows that the total delaies of two HERO algorithms are better than that of the MobySpace algorithms.

Figure 11 and Figure 12 show that in terms of average relay latency and the average total latency, Mobyspace has the worst performance while Epidemic has the best performance, and the performances of HERO algorithms are average.

Additionally, with the increase of *U*, the relay latencies and total latencies of the newly added successful communication pairs have a significant impact on the average relay latencies and average total latencies of these algorithms. As a result, their performance fluctuate with *U* increases.

**Figure 13:**
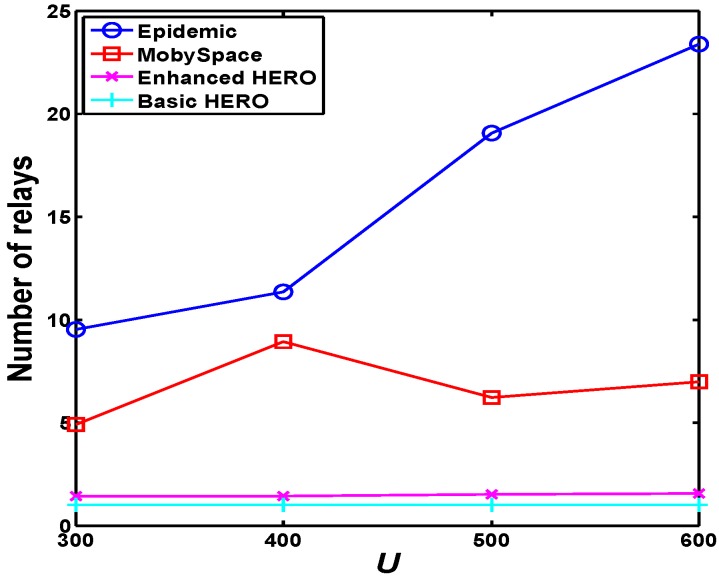
The average number of relays in the four schemes. Owing to the adopted single-copy mechanism, the relay numbers of HERO algorithms are less than those of Epidemic and Mobyspace algorithms.

Figure 13 reports the average number of relays employed by each of the four schemes. The number of relays in Epidemic is calculated by including all the relays employed before the first copy of the data arrives at the destination.

Figure 13 demonstrates the average relay number of the source-destination pairs that communicate successfully in these four schemes. The number of relays affect the transmission cost. The larger the number of relays, the higher the transmission cost.

From Figure 13, we see that the transmission costs of Epidemic and Mobyspace are respectively the highest and the second highest. The transmission cost of enhanced HERO is moderate, and the transmission cost of basic HERO is minimal because the basic algorithm seeks only one relay for the data delivery.

## 7. Conclusions

In this paper, two HERO algorithms called basic HERO and the enhanced HERO are proposed. The HERO algorithms make use of the spatial regularity of human mobility in relay selection. Both algorithms are single-copy algorithms, but the basic HERO is a single-hop algorithm while the enhanced HERO is a multi-hop algorithm. Both HERO algorithms are based on the concept of *home*. In the basic HERO algorithm, the relay is the first encountered node whose home includes the zone where the destination resides in. The enhanced HERO keeps finding a relay with high visiting intensity to the destination.

We use the mobility trace data of Dartmouth College to evaluate the performance of both HERO algorithms and compare them with MobySpace and Epidemic. We also provide a thorough theoretical analysis on our algorithms for the same set of metrics. The evaluation results show that the HERO algorithms outperform Mobyspace but are inferior to Epidemic, which provides an upper-bound on routing performance. However, the transmission cost of Epidemic is much higher than others.

Our future work will focus on the multi-copy variants of HERO that do not just simply apply the relay selection rules of the single copy versions proposed in this paper. In addition, we will design a social HERO relay selection framework that simultaneously considers the mobility and sociality characteristics of human beings.

## References

[1] Wang S., Liu M., Cheng X., Song M. (2012). Routing in Pocket Switched Networks. IEEE Wirel. Commun..

[2] Wang S., Liu M., Cheng X., Huang J., Chen B. (2013). Opportunistic Routing in Intermittently Connected Mobile P2P Networks. IEEE J. Sel. Areas Commun..

[3] Wang S., Wang X., Huang J., Bie R., Cheng X. (2015). Analyzing the potential of mobile opportunistic networks for big data applications. IEEE Netw..

[4] Wang S., Wang X., Cheng X., Huang J., Bie R. The tempo-spatial information dissemination properties of mobile opportunistic networks with Levy mobility. Proceedings of The 34th International Conference on Distributed Computing Systems, IEEE.

[5] Wang X., Lin Y., Zhao Y., Cai Z. A Double Pulse Control Strategy for Misinformation Propagation in Human Mobile Opportunistic Networks. Proceedings of The 10th International Conference on Wireless Algorithms, Systems, and Applications.

[6] Lindgren A., Doria A., Schelén O. (2003). Probabilistic routing in intermittently connected networks. ACM Sigmob. Mob. Comput. Commun. Rev..

[7] Dubois-Ferriere H., Grossglauser M., Vetterli M. Age matters: Efficient route discovery in mobile ad hoc networks using encounter ages. Proceedings of the 4th ACM International Symposium on Mobile Ad Hoc Networking & Computing, ACM.

[8] Spyropoulos T., Turletti T., Obraczka K. (2009). Routing in Delay-Tolerant Networks Comprising Heterogeneous Node Populations. IEEE Trans. Mob. Comput..

[9] Gao W., Cao G. On exploiting transient contact patterns for data forwarding in delay tolerant networks. Proceedings of the 18th IEEE International Conference on Network Protocols (ICNP).

[10] Jones E.P., Li L., Schmidtke J.K., Ward P.A. (2007). Practical Routing in Delay Tolerant Networks. IEEE Trans. Mob. Comput..

[11] Burgess J., Gallagher B., Jensen D., Levine B.N. MaxProp: Routing for Vehicle-Based Disruption-Tolerant Networks. Proceedings of the IEEE INFOCOM.

[12] Hui P., Crowcroft J., Yoneki E. (2011). Bubble rap: Social-based forwarding in delay-tolerant networks. IEEE Trans. Mob. Comput..

[13] Daly E.M., Haahr M. Social network analysis for routing in disconnected delay-tolerant manets. Proceedings of the 8th ACM International Symposium on Mobile Ad Hoc Networking and Computing.

[14] Dang H., Wu H. (2010). Clustering and cluster-based routing protocol for delay-tolerant mobile networks. IEEE Trans. Wirel. Commun..

[15] Liu C., Wu J. Scalable routing in delay tolerant networks. Proceedings of the 8th ACM International Symposium on Mobile Ad Hoc Networking and Computing.

[16] Costa P., Mascolo C., Musolesi M., Picco G.P. (2008). Socially-aware routing for publish-subscribe in delay-tolerant mobile ad hoc networks. IEEE J. Sel. Areas Commun..

[17] Gao W., Cao G. User-centric data dissemination in disruption tolerant networks. Proceedings of the IEEE INFOCOM.

[18] Mei A., Morabito G., Santi P., Stefa J. Social-aware stateless forwarding in pocket switched networks. Proceedings of the IEEE INFOCOM.

[19] Wu J., Wang Y. Social feature-based multi-path routing in delay tolerant networks. Proceedings of the IEEE INFOCOM.

[20] Leguay J., Friedman T., Conan V. Evaluating Mobility Pattern Space Routing for DTNs. Proceedings of the IEEE INFOCOM.

[21] Wu J., Xiao M., Huang L. Homing spread: Community home-based multi-copy routing in mobile social networks. Proceedings of the IEEE INFOCOM.

[22] Guo L., Zhang C., Yue H., Fang Y. A privacy-preserving social-assisted mobile content dissemination scheme in DTNs. Proceedings of the IEEE INFOCOM.

[23] Henderson T., Kotz D., Abyzov I., Yeo J. CRAWDAD Trace Set Dartmouth/Campus/Movement (v. 2005-03-08). http://crawdad.cs.dartmouth.edu/dartmouth/campus/20050308/.

[24] Gonzalez M.C., Hidalgo C.A., Barabasi A.L. (2008). Understanding individual human mobility patterns. Nature.

[25] McNett M., Voelker G.M. (2005). Access and mobility of wireless PDA users. ACM SIGMOBILE Mob. Comput. Commun. Rev..

[26] Trivedi K.S. (2002). Probability and Statistics With Reliability, Queuing and Computer Science Applications.

[27] Vahdat A., Becker D. (2000). Epidemic Routing for Partially Connected Ad Hoc Networks.

